# Associations Between Lower Extremity Myotonic Properties, Strength, and Balance in American Football Players: An Exploratory LASSO-Based Study

**DOI:** 10.3390/jcm15124842

**Published:** 2026-06-22

**Authors:** Derya Azim, Ömer Özer, Ahmet Kurtoğlu, Safaa M. Elkholi

**Affiliations:** 1 Faculty of Health Sciences, Department of Physiotherapy and Rehabilitation, Bandirma Onyedi Eylul University, 10200 Balikesir, Turkey; 2 Faculty of Sport Sciences, Department of Physical Education and Sport, Bandirma Onyedi Eylul University, 10200 Balikesir, Turkey; oozer@bandirma.edu.tr; 3 Faculty of Sport Science, Department of Coaching Education, Bandirma Onyedi Eylul University, 10200 Balikesir, Turkey; kurtogluahmet18@gmail.com; 4 Department of Rehabilitation Sciences, College of Health and Rehabilitation Sciences, Princess Nourah Bint Abdulrahman University, P.O. Box 84428, Riyadh 11671, Saudi Arabia

**Keywords:** myotonometry, muscle stiffness, strength, balance, sex differences, kinetic chain

## Abstract

**Background/Objectives:** Evidence on the role of muscle mechanical (myotonic) properties in athletic performance remains limited in young adult and sub-elite populations, particularly in American football, and sex-specific patterns of association are not well understood. This study aimed to investigate the associations between lower extremity myotonic properties and performance outcomes (strength and balance) in American football athletes, with a specific focus on sex-related differences and candidate predictors. **Methods:** A cross-sectional design was implemented involving 35 American football athletes (17 female, 18 male). Lower extremity muscle tone, stiffness, and elasticity were assessed using MyotonPRO. Strength parameters (lower limb, handgrip, back, and shoulder internal rotation) and balance performance (static and dynamic under eyes-open and eyes-closed conditions) were evaluated using standardized measurement protocols. Pearson correlation analysis was conducted to examine bivariate associations, followed by Least Absolute Shrinkage and Selection Operator (LASSO) regression to determine candidate predictors while addressing multicollinearity. **Results:** Male athletes exhibited significantly greater height, body mass, and BMI (*p* < 0.001), alongside elevated myotonic values compared to females. Correlation analyses indicated distinct sex-specific association patterns between myotonic properties and performance metrics. LASSO regression revealed a distinct sex-specific divergence in strength prediction: female strength was predominantly driven by proximal musculature (quadriceps and hamstring elasticity/stiffness), whereas male strength was anchored by distal musculature (gastrocnemius tone/stiffness). Furthermore, rigorous penalization shrunk nearly all balance coefficients to zero in both sexes, indicating that resting myotonic properties do not independently predict dynamic or static postural control. **Conclusions:** While lower extremity myotonic properties are candidate predictors of multi-regional strength via sex-specific proximal and distal strategies, they do not independently predict balance performance, suggesting postural control relies primarily on active motor recruitment rather than passive resting mechanics. Given the cross-sectional design of this study, causal inferences cannot be drawn, and these findings should be interpreted accordingly. The observed sex-specific differences may support consideration of individualized, sex-informed training strategies in American football athletes.

## 1. Introduction

Systematic monitoring of neuromechanical parameters has become increasingly relevant for optimizing athletic performance and informing evidence-based training prescription in American football [1]. The participants in the present study competed in [flag/limited-contact/tackle] American football, a format characterized by high lower-extremity mechanical demands, including rapid acceleration and deceleration, changes in direction, jumping, and single-leg balance tasks [2,3]. Although the magnitude and nature of contact exposure may differ across American football formats, lower-extremity neuromuscular function remains central to performance execution. Therefore, examining lower-extremity neuromuscular characteristics, including muscle mechanical properties, strength, and dynamic balance, may provide exploratory insight into performance-related profiles in American football athletes.

In this context, the assessment of lower extremity muscle mechanical properties provides valuable insight into the interaction between muscle stiffness, elasticity, and viscoelastic damping, which are key determinants of force production and dynamic stability [4]. These neuromuscular characteristics are particularly important in high-intensity sports, where repeated explosive actions and rapid force transmission are essential for performance [5].

Myotonometry enables the non-invasive quantification of muscle mechanical properties, including oscillation frequency (muscle tone), stiffness, and elasticity. These parameters reflect the intrinsic mechanical behavior of muscle tissue and have been proposed as potential correlates of neuromuscular function; however, it should be no-ted that resting MyotonPRO values reflect passive mechanical behavior and do not directly measure neuromuscular readiness or dynamic force transmission. Previous studies have demonstrated that muscle stiffness is associated with performance outcomes such as sprinting, maneuverability, and jumping ability, highlighting its functional relevance in dynamic athletic tasks [5]. However, the relationship between these mechanical properties and functional performance outcomes, particularly strength and balance, remains complex and not fully elucidated. In this regard, the MyotonPRO device has demonstrated good-to-excellent test–retest reliability (ICC > 0.80) across various muscle groups and athletic populations, supporting its suitability for repeated assessments in sports science research. Its portability, non-invasiveness, and speed of measurement further enhance its practical relevance in field-based and laboratory settings, enabling efficient neuromuscular profiling of athletes without disrupting training schedules [6,7].

Balance performance is closely linked to lower extremity neuromuscular function, as effective postural control requires coordinated interaction between muscle mechanical properties and force-generating capacity. In particular, dynamic balance depends on the ability to rapidly modulate muscle stiffness and elasticity in response to external perturbations. Despite this, existing research examining the relationship between muscle mechanical properties, strength, and balance has predominantly focused on elite athletes or non-athletic populations, with limited data available for young adult athletic cohorts [8]. Furthermore, whether cross-sectional, resting (passive) myotonic assessments can adequately capture the complex, active neural demands of dynamic postural control remains an important methodological question.

Biological sex is another important factor influencing neuromuscular performance. Male and female athletes exhibit distinct biomechanical strategies, muscle activation patterns, and structural characteristics, which may affect both force production and balance control [9]. For example, female athletes have been reported to demonstrate superior static balance, whereas male athletes may rely more heavily on force-oriented strategies during dynamic tasks [4]. These findings suggest that the interaction between muscle mechanical properties and performance outcomes may vary between sexes.

Additionally, imbalances between muscle strength and tendon stiffness can increase mechanical load on the tendon and elevate injury risk, underscoring the importance of evaluating muscle–tendon unit behavior in athletic populations [9]. Variability in muscle strength and tendon strain during high-intensity contractions further highlights the complexity of musculotendinous adaptation and its implications for performance and injury prevention [10]. Therefore, the aim of the present study was to investigate the relationships between lower extremity muscle mechanical properties-specifically oscillation frequency, stiffness, and elasticity-and measures of strength and balance in young American football athletes, with a particular focus on sex-based differences.

Based on the existing literature, we hypothesized that (1) lower extremity myotonic properties would be associated with strength and balance performance outcomes, (2) distal muscles would emerge as stronger candidate predictors of performance compared to proximal muscles, and (3) the nature and magnitude of these associations would differ between male and female athletes, reflecting sex-specific neuromuscular characteristics.

## 2. Materials and Methods

### 2.1. Study Design and Participants

This cross-sectional study aimed to investigate the relationships between lower extremity muscle mechanical properties, strength, and balance performance in young American football athletes, with a specific focus on sex-based differences.

A total of 35 athletes (female: n = 17; male: n = 18) voluntarily participated in the study. A priori sample size estimation was conducted using G*Power (version 3.1) based on Pearson correlation analysis, with a medium effect size (r = 0.46), α = 0.05, and power of 0.80, which indicated a minimum required sample of 34 participants (Actual Power = 80%). The recruited sample of 35 athletes exceeded this threshold. It should be noted, however, that this power estimate was based solely on bivariate Pearson correlation and did not account for the multiple sex-specific LASSO regression models subsequently performed. No formal power analysis was conducted for the LASSO analyses or for the sex-stratified subgroups (n = 17 females, n = 18 males). Consistent with the exploratory framing stated in the title of this study, the authors explicitly acknowledge that the sex-stratified subgroup sizes (n = 17 females, n = 18 males) impose meaningful constraints on the stability of the LASSO-derived coefficients, given the large number of myotonic variables examined relative to the number of observations. Accordingly, a post hoc sensitivity analysis was conducted using G*Power (version 3.1) to characterize the minimum detectable effect size under the present conditions (linear multiple regression, fixed model; α = 0.05; power = 0.80; n = 17–18 per subgroup). The results indicated that the available subgroup sizes support adequate power only for large effect sizes (f^2^ ≥ 0.35), which are larger than those typically observed in myotonometric research. Participants were recruited from local American football teams according to predefined inclusion and exclusion criteria. All athletes were actively competing in the same American football format and were exposed to comparable sport-specific training and competition demands. The sample consisted of young adult, sub-elite American football athletes who trained regularly and participated in organized team-based practice and competition. Male and female athletes were drawn from the same competitive environment and were assessed using identical testing procedures, thereby reducing methodological heterogeneity between sex groups. Although no restriction was applied according to playing position, all participants were involved in the same sport-specific performance context, including repeated acceleration and deceleration, changes in direction, jumping, and balance-related tasks. These characteristics were specified to clarify the competitive level, sport format, and relative homogeneity of the study population, and to support appropriate interpretation of the generalizability of the findings.

Individuals aged between 18 and 30 years who had at least one year of regular participation in American football and engaged in training at least three times per week were included in the study. All participants were required to be free from pain or injury in the lower extremities at the time of assessment and to provide written informed consent prior to participation.

Participants were excluded if they had a history of lower extremity injury or surgical intervention within the previous three months, any vestibular, neurological, or visual disorder that could affect balance, or any chronic musculoskeletal condition. Additional exclusion criteria included a body mass index (BMI) greater than 35 kg/m^2^, regular use of muscle relaxant or analgesic medications, and pregnancy.

All participants provided written informed consent prior to participation. Approval for this study was granted by the Non-Interventional Ethics Committee of Bandırma Onyedi Eylül University Faculty of Health Sciences (date number: 17 November 2025, number: 2025-33) and conducted in accordance with the Declaration of Helsinki.

### 2.2. Experimental Procedures

All assessments were conducted in a controlled laboratory environment under standardized conditions, including stable ambient temperature and minimal external noise, to ensure consistency across participants. Participants were instructed to refrain from strenuous physical activity for at least 24 h prior to testing. Upon arrival, demographic information, including age, body mass index (BMI), dominant limb, and training history, was recorded. Among the recruited participants, the majority of both female and male athletes reported right limb dominance (females: n = 15 right, n = 2 left; males: n = 16 right, n = 2 left).

Testing procedures were performed in a sequential order to minimize potential fatigue effects. First, muscle mechanical properties of the lower extremity were assessed using myotonometry. Following this, participants underwent balance assessment, and finally, strength measurements were performed. This order was selected so that passive myotonometry would not be influenced by prior physical exertion, and so that balance testing would precede the higher-demand maximal strength assessments. A standardized 5-min low-intensity cycling warm-up was completed by all participants before the balance and strength assessments but not before myotonometry, in order to preserve resting muscle conditions for the mechanical property measurements. A rest interval of approximately 3 min was provided between the balance and strength testing blocks, and 60 s of rest was allowed between individual trials within each strength test. It is acknowledged that the fixed testing order represents a limitation, as cumulative fatigue from balance testing may have influenced subsequent strength measurements. However, given the passive nature of myotonometry and the low physical demand of balance testing relative to maximal strength efforts, the potential confounding effect is considered minimal. Future studies may consider counterbalancing the order to formally assess order effects.

All measurements were carried out by the same experienced researcher to ensure consistency and reduce inter-rater variability. Participants were provided with standardized instructions before each assessment, and familiarization trials were conducted when necessary to ensure proper execution of the tests. To further enhance reproducibility, all myotonometry measurements were performed with participants in a standardized supine position with muscles at rest, and anatomical landmarks were marked on the skin to ensure consistent probe placement across measurements. All testing was conducted between 09:00 and 13:00 to control for potential diurnal variation in muscle mechanical properties and neuromuscular performance.

### 2.3. Outcome Measures

Lower extremity muscle mechanical properties, balance performance, and muscle strength were assessed as the primary outcome measures.

Muscle mechanical properties were evaluated using a handheld myotonometry device (MyotonPRO, Myoton AS, Tallinn, Estonia). Measurements were obtained bilaterally from the gastrocnemius medialis, gastrocnemius lateralis, tibialis anterior, quadriceps (rectus femoris), and hamstring muscles. The device applies a brief mechanical impulse to the muscle, generating oscillations that are used to quantify oscillation frequency (Hz), representing muscle tone, stiffness (N/m), reflecting resistance to deformation, and elasticity (logarithmic decrement), indicating the ability of the muscle to return to its original shape [11]. Three consecutive measurements were performed at each site, and the mean value was used for analysis. Measurements were performed prior to any warm-up activity to ensure resting muscle conditions were maintained. All participants were in a standardized supine position with muscles fully relaxed throughout the assessment. Anatomical landmarks for probe placement were as follows: gastrocnemius medialis—the most prominent point of the medial belly, midway between the popliteal crease and the medial malleolus; gastrocnemius lateralis—the most prominent point of the lateral belly at the same longitudinal level; tibialis anterior—one-third of the distance from the tibial tuberosity to the medial malleolus, lateral to the tibial crest; quadriceps (rectus femoris)—midpoint of the line from the anterior superior iliac spine to the superior border of the patella; hamstrings (biceps femoris)—midpoint of the posterior thigh between the ischial tuberosity and the lateral epicondyle of the femur. These sites were marked with a skin-safe marker prior to measurement and verified before each trial. Measurements proceeded from the dominant to the non-dominant limb in a fixed order for all participants. The assessor was not blinded to participant sex; however, all myotonometry measurements were completed before any performance scores were available, thereby preventing outcome knowledge from influencing probe placement or data recording. To assess intra-rater reliability of the MyotonPRO measurements in this sample, the coefficient of variation (CV) across the three consecutive trials at each site was calculated. Mean CV values were below 5% across all muscle sites and conditions, consistent with the acceptable threshold reported in the literature and confirming the repeatability of the measurement protocol.

Balance performance was assessed using the Korebalance™ Premiere system (Med-Fit Systems, Inc., Independence, VA, USA). Korebalance™ Premiere is a computerized balance platform designed to assess balance and deliver balance training. The system consists of a platform mounted on a variable air-pressure cushion and equipped with a tilt sensor (solid-state accelerometer/tilt sensor). This configuration enables detection of body sway within a 360° horizontal plane and approximately 20° of vertical inclination, with real-time data transmitted to dedicated computer software. Korebalance™ Premiere quantitatively and reproducibly evaluates both static and dynamic balance through its air-supported, sensor-integrated platform and software interface. Empirical studies indicate that the system demonstrates moderate agreement with widely used clinical balance tests and exhibits high reliability [12,13]. Static and dynamic balance were evaluated under both eyes open (EO) and eyes closed (EC) conditions. The recorded parameters included EO static balance, EO dynamic balance, EC static balance, and EC dynamic balance total scores. Intra-session reliability of the Korebalance system has been reported as good to excellent in published studies (ICC = 0.75–0.94 across static and dynamic conditions), supporting its use in the present protocol. For all balance conditions, the total score reflects cumulative body sway; higher scores indicate greater postural instability (i.e., worse balance performance), whereas lower scores reflect better postural control.

Muscle strength was assessed using dynamometric methods. Hand grip strength was measured using a Takei dynamometer (T.K.K.5401 GRIP-D, Takei Scientific Instruments Co., Ltd., Tokyo, Japan), which was adjusted according to the participant’s hand size. The participant was instructed to hold the shoulder in approximately 45° of abduction and to squeeze the dynamometer with maximal effort. Two trials were performed for each hand, and the highest value was recorded in kilograms (kg) [14].

Back strength was assessed using a Takei dynamometer. The participant stood with the knees fully extended and was instructed to exert maximal force by pulling the dynamometer vertically, with the load applied to the back region. Two trials were performed, and the highest value was recorded in kilograms (kg) [15].

Lower extremity maximal strength was assessed using a Takei dynamometer. The participant maintained a slight knee flexion position and was instructed to apply maximal force by pulling the dynamometer vertically, with the load directed to the lower extremities. Two trials were conducted, and the highest value was recorded in kilograms (kg) [16]. The same procedure was also performed unilaterally for the lower extremities. The participant completed the test in a single-leg stance for both the right and left legs separately, maintaining slight knee flexion while exerting maximal vertical force on the dynamometer. Two trials were conducted for each leg, and the highest values were recorded in kilograms (kg).

Isometric upper extremity strength was measured using a wireless handheld dynamometer (ActivForce 2, ActivBody Inc., San Diego, CA, USA). Participants stood next to a fixed door frame with the tested limb positioned in a sport-specific throwing posture (shoulder in abduction and external rotation; elbow at 135° flexion, verified by goniometer). The device was secured to the palm and placed against the door frame. After a standardized warm-up, participants performed three maximal voluntary isometric contractions (MVICs), each lasting 5 s, with 60 s of rest between trials. The highest value was recorded in kilograms (kg) [17]. All measurements were conducted by the same investigator. Shoulder internal rotation strength was included as a sport-relevant upper extremity performance parameter given the throwing and blocking demands of American football, and to enable exploratory examination of possible kinetic chain associations between lower extremity mechanical properties and upper body force output. Its inclusion does not imply a mechanistic relationship, and all such associations are interpreted observationally.

### 2.4. Statistical Analysis

All statistical analyses and data visualizations were performed using the R programming environment (v4.3.0, R Core Team, Vienna, Austria). Continuous variables were presented as mean ± standard deviation (SD). The assumption of normality for data distribution was assessed using the Shapiro–Wilk test and visual inspection of Q-Q plots. To evaluate baseline demographic and functional differences between female and male athletes, an independent samples *t*-test was conducted. For all sex-stratified analyses, the final sample consisted of 17 females and 18 males. Bilateral measurements (left and right sides) were aggregated by calculating the mean value for each parameter to ensure a single representative data point per participant, thereby avoiding the inflation of sample size and potential pseudo-replication. There were no missing data points in the analyzed dataset. The analytical framework for the primary outcomes was designed in a two-stage complementary approach to map both the general associations and the candidate predictors of strength and balance parameters. Initially, Pearson’s correlation coefficients were calculated to explore the bivariate relationships between the resting myotonic properties of lower extremity muscles and the functional target variables, aiming to map the general myofascial interaction network. These bivariate correlation analyses were strictly exploratory. Therefore, uncorrected *p*-values were reported and should be interpreted cautiously, acknowledging the inherent risk of Type I error inflation and potential false positives due to multiple comparisons. However, to resolve the inherent multicollinearity among functionally coupled muscle groups and to identify the true candidate predictors of strength and balance, a Least Absolute Shrinkage and Selection Operator (LASSO) regression model was employed using the glmnet (v4.1-8) package. For each target performance parameter, a total of 30 myotonic predictors were entered into the model simultaneously. Prior to model fitting, all predictor variables were internally standardized to ensure uniform penalization. LASSO applies an L1-regularization penalty that shrinks the regression coefficients of less contributive or redundant variables exactly to zero, effectively performing simultaneous feature selection and model regularization. For each target parameter, the optimal penalization factor (λ) was determined using a 5-fold cross-validation procedure to mitigate the risk of unstable lambda selection due to the relatively small sample sizes. The λ value that minimized the mean cross-validated error was selected to extract the final non-zero coefficients, which were deemed significant candidate predictors without the reliance on traditional *p*-values. Finally, comprehensive data visualizations, including traditional correlation heatmaps and faceted sparse coefficient matrices for LASSO results, were generated using the ggplot2 (v3.4.4) package to ensure high interpretability of the multidimensional findings. The statistical significance level for all non-penalized initial tests was set at *p* < 0.05 a priori. Given the relatively small sex-stratified subgroup sizes (n = 17 females, n = 18 males) and the exploratory nature of the analyses, all findings-particularly those from the sex-specific Pearson correlations and LASSO models-should be regarded as hypothesis-generating rather than confirmatory. Furthermore, because bootstrap stability selection was not performed to evaluate how often each predictor was retained across resampling, the stability of the selected features cannot be definitively guaranteed. Consequently, the selected LASSO predictors must be interpreted very cautiously.

## 3. Results

In the examination of demographic characteristics and training habits of male and female participants of the research study, no statistically significant difference between the two groups in terms of age (*p* = 0.131), sporting experience (*p* = 0.190), weekly training frequency (*p* = 0.086), and weekly training duration (*p* = 0.174) was observed. However, in the examination of physical characteristics of the participants, it was observed that the height of the male participants (180.12 ± 5.94 cm), their weight (81.24 ± 9.10 kg), and BMI values (25.03 ± 2.41 kg/m^2^) were statistically significantly higher than the female participants (*p* < 0.001) (Table 1). This suggests that although the two groups have different physical structures, they have similar characteristics in terms of sporting history and training intensity.

Male athletes exhibited significantly higher resting myotonic tone and stiffness across all measured lower extremity muscles, as well as superior upper and lower body strength parameters, compared to female athletes, with predominantly large effect sizes. Detailed descriptive statistics, between-sex comparisons, and effect sizes (Cohen’s d) for all myotonic, strength, and balance variables are provided in Supplementary Table S1.

**Figure 1:**
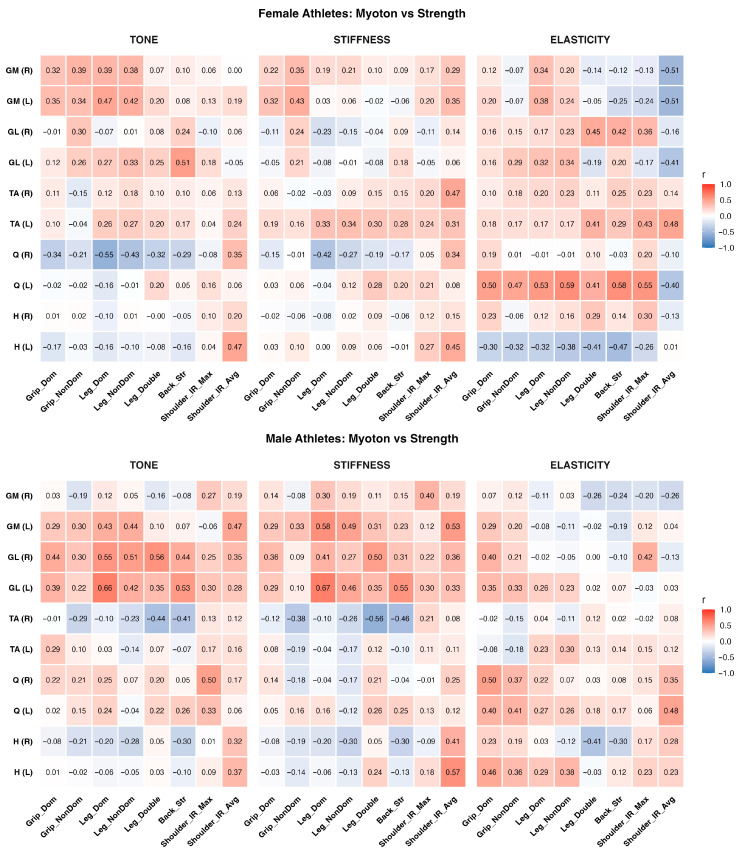
Heatmap correlation matrix of myotonometric parameters and muscle strength variables.

The bivariate relationships between the resting myotonic properties of the lower limb muscles and various strength parameters in female athletes (n = 17) were examined using Pearson correlation coefficients (Figure 1). The analysis revealed that specific mechanical properties of the lower extremity were significantly correlated with global force production. Notably, left Quadriceps elasticity emerged as a prominent predictor across multiple kinetic chain segments, demonstrating significant positive correlations with dominant handgrip strength (r = 0.499, *p* = 0.041), dominant leg strength (r = 0.530, *p* = 0.028), non-dominant leg strength (r = 0.591, *p* = 0.012), back strength (r = 0.581, *p* = 0.014), and maximal shoulder internal rotation (r = 0.551, *p* = 0.021). In addition, right Quadriceps tone was negatively correlated with dominant leg strength (r = −0.554, *p* = 0.020). Furthermore, significant associations were observed in the distal musculature; right Gastrocnemius Medialis (GM) elasticity correlated negatively with average shoulder internal rotation (r = −0.512, *p* = 0.035), while left Gastrocnemius Lateralis (GL) tone correlated positively with back strength (r = 0.512, *p* = 0.035). Other examined parameters of muscle tone, stiffness, and elasticity did not demonstrate statistically significant bivariate relationships with the remaining strength outcomes (*p* > 0.05).

The bivariate relationships between the resting myotonic properties of the lower limb muscles and strength parameters in male athletes (n = 18) were assessed using Pearson correlation coefficients (Figure 1). Following data aggregation to ensure strictly independent observations, several robust and statistically significant correlations emerged. Lower extremity strength was predominantly associated with the mechanical properties of the gastrocnemius muscle. Specifically, dominant leg strength demonstrated strong positive correlations with left Gastrocnemius Lateralis (GL) stiffness (r = 0.667, *p* = 0.003) and tone (r = 0.655, *p* = 0.003), as well as left Gastrocnemius Medialis (GM) stiffness (r = 0.580, *p* = 0.012). Furthermore, right GL tone consistently and positively correlated with dominant leg strength (r = 0.548, *p* = 0.019), non-dominant leg strength (r = 0.506, *p* = 0.032), and double leg strength (r = 0.561, *p* = 0.015). Conversely, right Tibialis Anterior (TA) stiffness exhibited a significant negative correlation with double leg strength (r = −0.563, *p* = 0.015). Regarding trunk performance, back strength was significantly and positively correlated with left GL stiffness (r = 0.553, *p* = 0.017) and tone (r = 0.529, *p* = 0.024). For upper extremity parameters, dominant handgrip strength correlated positively with right Quadriceps (Q) elasticity (r = 0.501, *p* = 0.034). Finally, shoulder internal rotation performance was linked to multiple myotonic profiles; maximal rotation correlated positively with right Q tone (r = 0.499, *p* = 0.035), while average rotation demonstrated significant positive associations with left Hamstring stiffness (r = 0.574, *p* = 0.013), left GM stiffness (r = 0.525, *p* = 0.025), and left Q elasticity (r = 0.481, *p* = 0.043). Other examined parameters did not reach statistical significance (*p* > 0.05).

**Figure 2:**
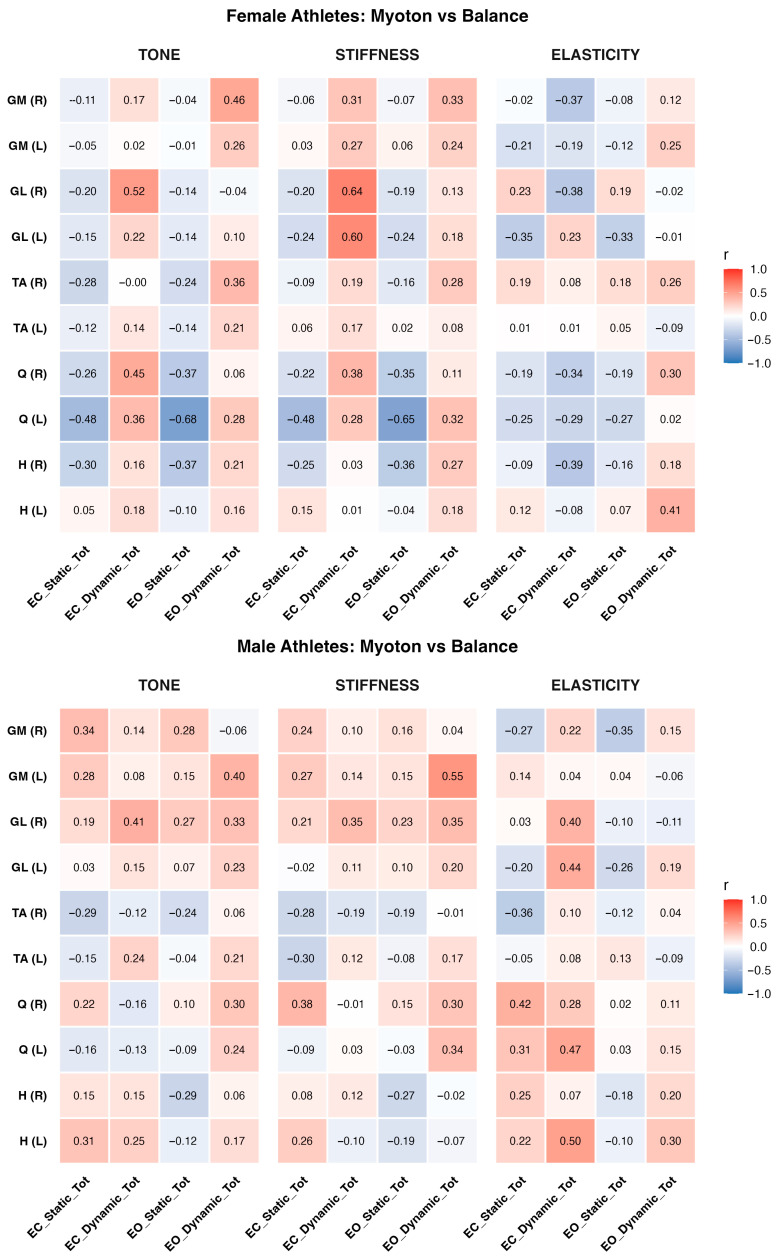
Heatmap correlation matrix of myotonometric parameters and balance variables.

The relationship between the resting myotonic properties of the lower extremity muscles and static and dynamic balance parameters in female athletes (n = 17) was evaluated using Pearson correlation coefficients (Figure 2). Balance was expressed as total sway scores under Eyes Closed (EC) and Eyes Open (EO) conditions, where higher scores indicate greater postural sway (i.e., poorer balance). Following data aggregation to isolate independent observations, the corrected analysis revealed highly specific, condition-dependent relationships. Under EC conditions, dynamic sway was significantly and positively correlated with right GL tone (r = 0.522, *p* = 0.031) and stiffness (r = 0.644, *p* = 0.005), as well as left GL stiffness (r = 0.605, *p* = 0.010), indicating that increased stiffness in the distal musculature is associated with poorer dynamic postural control when visual input is removed. Conversely, left Q tone exhibited a significant negative correlation with EC static sway (r = −0.483, *p* = 0.050). Under EO conditions, static sway demonstrated strong negative correlations with both left Q tone (r = −0.681, *p* = 0.003) and left Q stiffness (r = −0.654, *p* = 0.004), suggesting that higher tone and stiffness in the proximal anterior musculature are associated with enhanced static postural control when visual feedback is available. No other statistically significant relationships were observed between the remaining myotonic properties and balance outcomes (*p* > 0.05).

Similarly, Pearson correlation analysis was conducted to assess the relationship between myotonic characteristics and postural sway parameters in male athletes (n = 18) (Figure 2). The corrected analysis identified distinct, task-specific associations primarily linked to dynamic stability. Under EO conditions, dynamic sway exhibited a statistically significant positive correlation with left GM stiffness (r = 0.551, *p* = 0.018), indicating that increased medial calf stiffness is associated with greater dynamic instability. For EC conditions, dynamic sway demonstrated significant positive correlations with both left Q elasticity (r = 0.472, *p* = 0.048) and left H elasticity (r = 0.496, *p* = 0.036). The remaining myotonic parameters, including muscle tone and stiffness across other examined regions, did not demonstrate statistically significant associations with either static or dynamic balance outcomes (*p* > 0.05).

**Figure 3:**
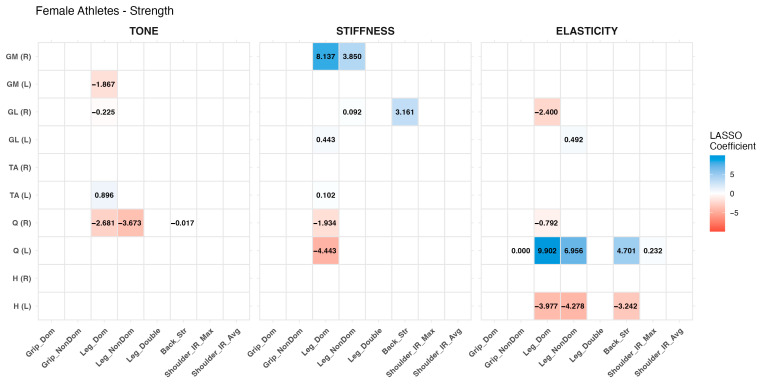
Heat map of LASSO regression coefficients identifying myotonic correlates of various strength parameters in females: GM: Gastrocnemius Medialis; GL: Gastrocnemius Lateralis; TA: Tibialis Anterior; Q: Quadriceps; H: Hamstrings; (R): Right; (L): Left, Grip_Dom: Dominant Handgrip Strength; Grip_NonDom: Non-dominant Handgrip Strength; Leg_Dom: Dominant Leg Strength; Leg_NonDom: Non-dominant Leg Strength; Leg_Double: Double Leg Strength; Back_Str: Back Strength; Shoulder_IR_Max: Maximal Shoulder Internal Rotation; Shoulder_IR_Avg: Average Shoulder Internal Rotation.

In Figure 3, the cross-validated LASSO regression analysis of the female participants identifies the true independent myotonic predictors of various strength parameters. The model results identified specific, highly regularized associations, highlighting the dominant predictive capacity of the proximal musculature. Regarding lower extremity performance, dominant leg strength (R^2^ = 0.967) showed the strongest positive associations with left Q elasticity (coefficient = 9.902) and right GM stiffness (coefficient = 8.137), whereas the strongest negative associations were observed for left Q stiffness (coefficient = −4.443) and left H elasticity (coefficient = −3.977). Similarly, non-dominant leg strength (R^2^ = 0.755) was most strongly positively predicted by left Q elasticity (coefficient = 6.956) and right GM stiffness (coefficient = 3.850), with left H elasticity (coefficient = −4.278) and right Q tone (coefficient = −3.673) acting as negative predictors.

In the case of trunk measurements, back strength (R^2^ = 0.536) depended mostly on the elasticity of left Q (coefficient = 4.701) and the stiffness of right GL (coefficient = 3.161), with the latter showing a negative correlation with the elasticity of left H (coefficient = −3.242). For the upper extremity performance, maximal shoulder internal rotation (R^2^ = 0.094) showed a positive relationship with the elasticity of left Q only (coefficient = 0.232). In particular, the tough L1 penalty of the LASSO regression shrank the coefficient for the dominant and non-dominant handgrip strength, double leg strength, and average shoulder internal rotation to zero. That is, in light of strict cross-validation and the presence of multicollinearity, resting myotonic parameters do not act as reliable candidate predictors for the specified force measures for females.

Combining all of the above findings, it can be concluded that the multi-predictor nature of the penalized LASSO model points out that myotonic variables do not depend exclusively on the local muscles being tested. Considering the data from a holistic perspective, the resting mechanical profile of the proximal lower extremity, in particular, elasticity and stiffness of the Q and H complex, can be seen as the key functional indicator revealing general neuromuscular characteristics and performance in females. Nevertheless, it is important to stress that the observed relationship does not represent evidence of the existence of general force transmission since all assessments were conducted at rest.

**Figure 4:**
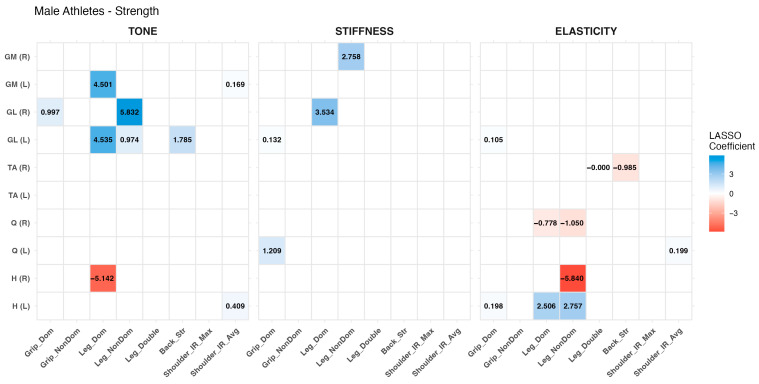
Heat map of LASSO regression coefficients identifying myotonic correlates of various strength parameters in males: GM: Gastrocnemius Medialis; GL: Gastrocnemius Lateralis; TA: Tibialis Anterior; Q: Quadriceps; H: Hamstrings; (R): Right; (L): Left, Grip_Dom: Dominant Handgrip Strength; Grip_NonDom: Non-dominant Handgrip Strength; Leg_Dom: Dominant Leg Strength; Leg_NonDom: Non-dominant Leg Strength; Leg_Double: Double Leg Strength; Back_Str: Back Strength; Shoulder_IR_Max: Maximal Shoulder Internal Rotation; Shoulder_IR_Avg: Average Shoulder Internal Rotation.

In the male participants, cross-validated LASSO regression analysis (Figure 4) showed a markedly distinct pattern, shifting the predictors from proximal muscles to distal lower extremity muscles. The dominant leg strength (R^2^ = 0.742) was predicted by the positive association with left GL tone (coefficient = 4.535), left GM tone (coefficient = 4.501), right GL stiffness (coefficient = 3.534), and left H elasticity (coefficient = 2.506), with a negative association with right H tone (coefficient = −5.142) and right Q elasticity (coefficient = −0.778). Non-dominant leg strength (R^2^ = 0.654) was characterized by positive associations with right GL tone (coefficient = 5.832), right GM stiffness (coefficient = 2.758), left H elasticity (coefficient = 2.757), and left GL tone (coefficient = 0.974), together with negative associations with right H elasticity (coefficient = −5.840) and right Q elasticity (coefficient = −1.050).

Concerning the measures of trunk performance, back strength (R^2^ = 0.254) had a positive association with left GL tone (coefficient = 1.785), whereas the association with right TA elasticity was negative (coefficient = −0.985). In regard to the upper extremity parameters, the dominant handgrip strength (R^2^ = 0.315) was predicted by left Q stiffness (coefficient = 1.209) and right GL tone (coefficient = 0.997), and smaller positive coefficients were found for left H elasticity (coefficient = 0.198), left GL stiffness (coefficient = 0.132), and left GL elasticity (coefficient = 0.105). The average shoulder internal rotation (R^2^ = 0.301) was positively related to left H tone (coefficient = 0.409), left Q elasticity (coefficient = 0.199), and left GM tone (coefficient = 0.169). Again, consistent with the conservative nature of the penalized model, the coefficients for the non-dominant handgrip strength, double leg strength, and maximal shoulder internal rotation were set to zero.

From an explorative point of view, the use of distal calf parameters as main predictors for upper body and trunk strength parameters is in line with the kinetic chain concept, indicating the sex-specific requirement of distal anchoring mechanism for force generation. Similar to the female group, these cross-sectional results are related to the resting mechanical profiles of subjects.

**Figure 5:**
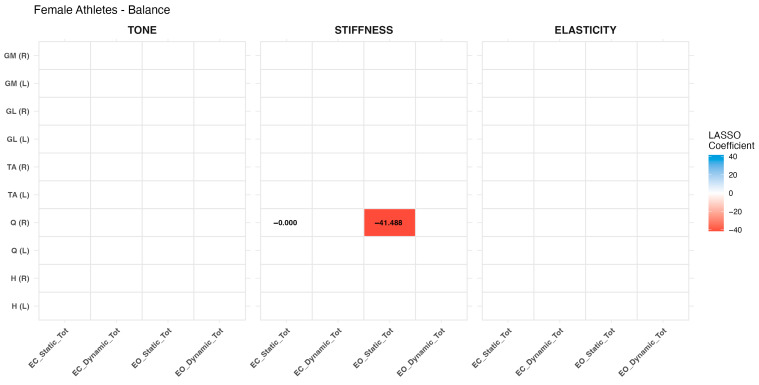
Heat map of LASSO regression coefficients identifying myotonic correlates of various balance parameters in females: EC_Static_Tot: Eyes Closed Static Total Sway Score; EC_Dynamic_Tot: Eyes Closed Dynamic Total Sway Score; EO_Static_Tot: Eyes Open Static Total Sway Score; EO_Dynamic_Tot: Eyes Open Dynamic Total Sway Score; GM: Gastrocnemius Medialis; GL: Gastrocnemius Lateralis; TA: Tibialis Anterior; Q: Quadriceps; H: Hamstrings; (R): Right; (L): Left.

Figure 5 illustrates the cross-validated LASSO regression analysis evaluating the independent myotonic predictors of static and dynamic balance parameters in female athletes. In stark contrast to the strength models, the algorithms predicting postural control demonstrated highly conservative behavior. The rigorous L1 penalization shrunk the coefficients for almost all balance conditions—specifically EC_Static_Tot, EC_Dynamic_Tot, and EO_Dynamic_Tot—entirely to zero. This zero-shrinkage indicates that resting muscle tone, stiffness, and elasticity are not robust, independent determinants of dynamic or visually deprived balance tasks in this cohort.

The only condition that survived the cross-validated penalization was EO_Static_Tot (R^2^ = 0.199), which exhibited a single, massive negative association with Q (R) stiffness (coefficient = −41.488). Given that lower sway scores indicate superior balance, this substantial negative coefficient suggests that higher resting stiffness in the right proximal anterior musculature independently predicts reduced sway when visual feedback is available and the base of support is static.

From a physiological perspective, this near-total shrinkage across the models is a critical finding. It explicitly demonstrates that while resting mechanical properties (particularly of the Q complex) may provide a passive structural anchor during simple, visually guided static tasks, they lack the predictive capacity for more complex postural demands. These results strongly suggest that dynamic and visually deprived balance strategies are governed primarily by active central nervous system motor unit recruitment and rapid active force generation, rather than the resting passive mechanics of the lower extremity.

**Figure 6:**
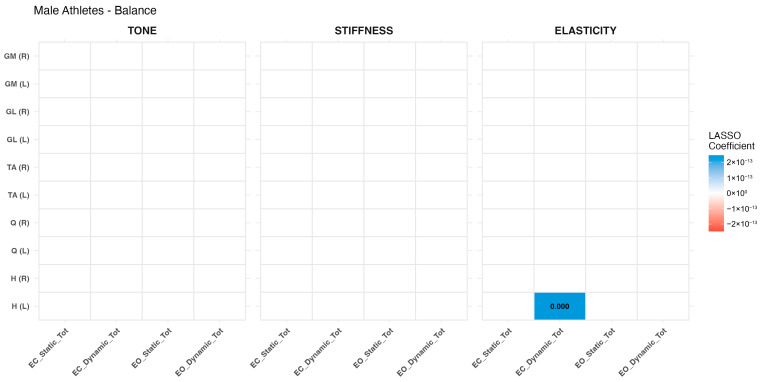
Heat map of LASSO regression coefficients identifying myotonic correlates of various balance parameters in males: EC_Static_Tot: Eyes Closed Static Total Sway Score; EC_Dynamic_Tot: Eyes Closed Dynamic Total Sway Score; EO_Static_Tot: Eyes Open Static Total Sway Score; EO_Dynamic_Tot: Eyes Open Dynamic Total Sway Score; GM: Gastrocnemius Medialis; GL: Gastrocnemius Lateralis; TA: Tibialis Anterior; Q: Quadriceps; H: Hamstrings; (R): Right; (L): Left.

Figure 6 illustrates the cross-validated LASSO regression analysis evaluating the independent myotonic predictors of static and dynamic balance parameters in male athletes. The results demonstrated an extremely conservative predictive behavior. The rigorous L1 penalization of the algorithm shrunk the coefficients for all thirty candidate myotonic predictors across every visual and dynamic balance condition (EC_Static_Tot, EC_Dynamic_Tot, EO_Static_Tot, and EO_Dynamic_Tot) entirely to zero. Consequently, the explained variance (R^2^) for all male balance models was zero.

This complete zero-shrinkage explicitly indicates that resting muscle tone, stiffness, and elasticity do not serve as independent determinants of balance in this cohort. Physiologically, this profound lack of predictive capacity supports the paradigm that postural control is a strictly active, dynamic process. It relies on real-time central nervous system motor unit recruitment rather than the baseline passive mechanics of the lower extremity musculature. Therefore, the present data confirm that cross-sectional resting myotonic measurements cannot reliably infer dynamic postural strategies or balance performance in male athletes.

## 4. Discussion

The present study investigated the associations between lower extremity myotonic properties and performance outcomes in American football athletes, with a specific emphasis on sex-related differences. Addressing a notable gap in young adult and sub-elite populations [8], the findings suggest that resting muscle mechanical properties are associated with specific, sex-dependent strength profiles, but crucially, do not serve as candidate predictors for dynamic or static balance. This refines our understanding of their relevance as indicators of overall neuromuscular status.

The combined use of Pearson correlation and LASSO regression enabled both the identification of global association patterns and the isolation of variables showing the strongest independent associations while mitigating multicollinearity effects. This analytical framework aligns with previous recommendations for modeling complex neuromuscular systems [18]. The results indicate that performance outcomes are associated with multiple interacting mechanical muscle properties rather than isolated variables.

Sex-based differences were evident in both anthropometric characteristics and the pattern of associations between myotonic properties and performance. Males demonstrated higher values of muscle stiffness, tone, and elasticity, consistent with previous findings [19]. These differences reflect variations in muscle mass, fiber composition, and tendon stiffness, which are known to influence force production and mechanical efficiency [20]. In the broader literature, higher muscle-tendon stiffness has been reported in association with more efficient stretch–shortening cycle (SSC) utilization [1,21]; however, such mechanistic interpretations cannot be directly inferred from the resting measurements obtained in the present study. Conversely, lower stiffness profiles in females have been discussed in relation to certain musculoskeletal injury pattern [22,23]. Nevertheless, injury outcomes were not assessed in the present study, and therefore no inference regarding injury susceptibility can be drawn from the current data. It should also be noted that the male and female groups differed substantially in body mass (81.24 vs. 56.76 kg) and height (180.12 vs. 164.94 cm), both of which are established correlates of muscle mechanical properties and absolute strength. No covariate adjustment for these anthropometric differences was performed in the present analyses; therefore, it is possible that some or all of the observed sex-specific differences in myotonic properties and strength reflect body size rather than sex per se. Future studies should consider body-mass-normalized strength values (e.g., relative strength expressed per kilogram of body mass) and statistically control for anthropometric variables to disentangle the independent contributions of sex and body composition to the observed associations.

A key finding of this study is the pronounced sex-specific divergence in the myotonic predictors of strength. In female athletes, force production was predominantly predicted by the proximal musculature, specifically the elasticity and stiffness of the quadriceps and hamstring complex. Conversely, in male athletes, strength prediction was anchored by the distal lower extremity, primarily driven by gastrocnemius tone and stiffness. While the male distal profile aligns with certain kinetic chain perspectives [24], suggesting distal muscles may reflect broader performance-related characteristics, the female reliance on proximal structures highlights a distinctly different neuromuscular strategy. However, given that myotonic measurements were obtained at rest, these associations should be interpreted as reflecting general mechanical profiles rather than direct evidence of force transmission mechanisms.

Contrary to traditional bivariate assumptions, balance-related findings revealed a near-total absence of predictive associations. The rigorously penalized cross-validated LASSO models shrunk the coefficients for static and dynamic balance outcomes to zero across almost all conditions for both sexes. Rather than a methodological limitation, this zero-shrinkage is a highly revealing physiological finding. It explicitly demonstrates that resting, passive mechanical properties of the lower extremity do not dictate dynamic postural stability. Instead, these findings strongly support the paradigm that balance is a highly active process dependent on real-time neural processing, multisensory integration, and active motor unit recruitment, independent of baseline tissue stiffness. However, since muscle mechanical properties were not measured during active balance tasks, these findings should be interpreted as evidence against passive mechanical reliance rather than direct indicators of neuromuscular control strategies.

From a broader contextual perspective, the interaction between muscle strength and tendon stiffness has been discussed as a relevant factor for both performance and injury risk [9]. Reported sex-specific differences in post-injury stiffness adaptations [25,26] further underscore the importance of individualized assessment. In addition, differential responses of muscle and tendon tissues to mechanical loading [27] together with sex-specific adaptations to eccentric training [1] suggest that training-induced responses may vary substantially across individuals.

Neuromuscular training has been shown to reduce injury risk in athletes [28,29], and integrating myotonic profiling into such frameworks may further support individualized intervention strategies. Moreover, developmental factors, particularly in female young adult athletes, may influence neuromuscular adaptations and stretch–shortening cycle function [30]. However, because the present study was based on resting measurements, direct inferences regarding these dynamic neuromuscular processes cannot be made.

Emerging evidence also indicates that genetic factors, such as PIEZO1-related mechanisms, may influence tendon adaptation to loading [31]. Therefore, future studies should explore how these biological and training-related variables interact with myotonic properties. Longitudinal research examining training dose–response relationships is particularly needed [32], alongside investigations into sex- and maturation-specific neuromuscular adaptations [27].

Another important observation emerging from the present findings is the presence of side-specific differences in both strength and myotonic predictors, as reflected by the distinct roles of dominant and non-dominant limb variables in the LASSO models. In sports characterized by high levels of unilateral loading, such as football (e.g., kicking and passing), previous studies have shown that functional specialization may lead to pronounced asymmetries in strength and mechanical muscle properties between limbs [8,33]. In this context, the asymmetrical patterns observed in the current study appear to be consistent with bilateral differences in muscle mechanical properties and force production capacity. Indeed, systematic monitoring of such asymmetries has been emphasized as important for performance optimization and load management, particularly in elite athletes [34,35].

Although injury outcomes were not assessed in the present study, inter-limb asymmetries have been widely discussed as potential contributors to increased injury susceptibility [33,34]. Differences in strength and mechanical muscle properties between limbs may alter load distribution and promote compensatory movement patterns, which have been discussed in relation to musculoskeletal loading and injury susceptibility [36]. However, as these relationships were not directly examined in the current study, the present findings should be interpreted cautiously within an injury-risk framework.

In this context, the side-specific associations observed between myotonic properties and performance variables should not be interpreted as direct evidence of injury mechanisms, but rather as potential indicators of underlying neuromuscular imbalances [33,36]. Assessment tools such as myotonometry and tensiomyography may provide valuable insights into these asymmetries at the muscle level, supporting individualized training adjustments and rehabilitation strategies [37,38]. Therefore, while the present findings do not provide direct evidence of injury risk, they may contribute to identifying mechanical asymmetry profiles that are relevant within injury prevention frameworks.

This study has several limitations. First, myotonic properties were assessed under resting conditions, which limits direct interpretation of these variables during dynamic performance tasks. Second, the cross-sectional design precludes causal inference. Third, although the recruited sample exceeded the minimum threshold derived from the a priori power analysis, it is important to emphasize that this power estimate was based on bivariate Pearson correlation and was not specifically designed for the sex-stratified LASSO analyses. With only 17 female and 18 male participants, the sex-specific analyses are underpowered relative to the number of predictors examined, rendering them susceptible to unstable coefficient estimates, overfitting, and an elevated risk of false-positive findings. The relatively small sample size-particularly after stratification by sex-may have limited the stability of the LASSO-derived predictor selections and increased the risk of overfitting. While the regularization properties of LASSO reduce this concern, they do not eliminate it; therefore, the identified predictors should be interpreted as exploratory rather than confirmatory. While LASSO regression effectively manages high-dimensional data and multicollinearity, the relatively small sample sizes per sex group pose a mathematical limitation. Furthermore, bootstrap stability selection was not performed in our modeling pipeline. Because the frequency of predictor retention across multiple resamples was not assessed, the stability of the selected LASSO features cannot be definitively guaranteed. Consequently, the candidate predictors identified in this study should be interpreted cautiously and viewed as strictly exploratory and hypothesis-generating, warranting future validation in larger cohorts using stability selection protocols. Accordingly, the sex-specific Pearson correlations and LASSO models should be regarded as hypothesis-generating, and their findings should not be interpreted as confirmatory evidence of sex-specific neuromuscular relationships. Future studies with adequate sample sizes per sex are needed to validate these patterns. Fourth, although LASSO can reduce the impact of multicollinearity, residual confounding cannot be excluded. In particular, several potential confounding variables, including training history, sport-specific experience, maturity status, and body composition, were not systematically adjusted for in the analyses and may have influenced the observed associations. As noted in the Discussion, anthropometric differences between sexes (body mass and height) were not adjusted for as covariates; future studies employing body-mass-normalized strength values and covariate-adjusted models would help clarify the independent contribution of sex to the observed associations. Additionally, while dominant limb prevalence was recorded and was comparable across groups (females: 15 right, 2 left; males: 16 right, 2 left), limb dominance was not formally entered as a covariate in the bilateral analyses, which may account for a small portion of the side-specific variability observed. Although exploratory Pearson correlations were utilized to map general myofascial interactions, conducting multiple bivariate comparisons inherently increases the risk of Type I error inflation. To strictly mitigate this limitation and penalize redundant associations, our primary conclusions were drawn from the LASSO regression models, which isolate non-zero features optimized through cross-validation. Finally, the sample was limited to American football athletes, which may restrict the generalizability of the findings to other athletic populations. Future studies should incorporate longitudinal and dynamic measurement approaches to better understand the functional role of myotonic properties. Research focusing on developmental stages and sex-specific adaptations will further refine their application in performance and injury prevention contexts.

## 5. Conclusions

In this study, the resting mechanical characteristics of the lower extremities were associated with certain strength parameters depending on sex, yet notably not predicting postural stability performance independently from other strength parameters. This difference implies that while resting myotonic characteristics could help to predict certain force-generating capacities and mechanisms used by athletes in their kinetic chain, the postural control does not depend on these characteristics but rather depends on active neuroprocessing of information in the context of American football athletes. With regard to the explorative nature of the data obtained due to a cross-sectional design and measurements under resting conditions, this finding should be replicated in large samples in prospective studies.

Moreover, the differences between males and females observed in terms of which muscles were more influential when predicting maximal strength parameters imply distinct neuromuscular structures in these groups. Such information can be used in designing further research that can include sex-specific considerations and mechanical characteristics of athletes.

From a practical perspective, myotonic profiling can be included in comprehensive athlete monitoring procedures. The procedure itself is easy to perform, fast, and does not require special equipment beyond the MyotonPRO apparatus.

## Figures and Tables

**Table 1:** Comparison of Participants’ Demographic Characteristics by Gender.

Variable	Female	Male	*p*_Value	Cohen d
Age (year)	19.94 ± 1.48	21.06 ± 2.56	0.131	−0.57
Height (cm)	164.94 ± 5.52	180.12 ± 5.94	<0.001	−2.70
Weight (kg)	56.76 ± 6.94	81.24 ± 9.10	<0.001	−3.02
BMI (kg/m^2^)	20.85 ± 2.18	25.03 ± 2.41	<0.001	−1.79
Experience (years)	1.47 ± 0.72	2.53 ± 3.12	0.190	−0.43
Weekly Training Freq	4.76 ± 1.48	5.65 ± 1.37	0.086	−0.60
WTD (min)	111.76 ± 11.85	142.35 ± 88.00	0.174	−0.47

BMI: Body Mass Index; WTD: Weekly Training Duration.

## Data Availability

The data supporting the findings of this study are available from the corresponding author upon reasonable request. The data are not publicly available due to privacy and ethical restrictions.

## Supplementary Materials

The following supporting information can be downloaded at: https://www.mdpi.com/article/10.3390/jcm15124842/s1, Table S1: Descriptive statistics, between-sex comparisons, and effect sizes (Cohen’s d) for myotonic, strength, and balance variables; Table S2: Non-zero LASSO regression coefficients and model performance metrics by sex.

## Abbreviations

The following abbreviations are used in this manuscript:

BMI	Body Mass Index
EC	Eyes Closed
EO	Eyes Open
GM	Gastrocnemius Medialis
GL	Gastrocnemius Lateralis
TA	Tibialis Anterior
Q	Quadriceps (Rectus Femoris)
H	Hamstrings (Biceps Femoris)
R	Right
L	Left
