# Supplementary material for: Associations Between Lower Extremity Myotonic Properties, Strength, and Balance in American Football Players: An Exploratory LASSO-Based Study
Source: J Clin Med. 2026 Jun 22;15(12):4842. doi: 10.3390/jcm15124842 (PMC13301756; doi:10.3390/jcm15124842)
Supplement: Supplementary file 1 [file jcm-15-04842-s001.zip › jcm-4331994-supplementary/jcm-4331994-Table_S1.pdf]

Variable	Female (n=17)	Male (n=18)	p-value	Cohen's d
**Myotonic Properties**				
**GM (R) Tone**	14.77 ± 1.55	17.69 ± 1.34	< 0.001	-2.02
**GM (L) Tone**	14.44 ± 1.10	17.82 ± 1.47	< 0.001	-2.59
**GM (R) Stiff.**	255.47 ± 39.28	319.94 ± 38.18	< 0.001	-1.67
**GM (L) Stiff.**	250.29 ± 23.45	319.33 ± 38.01	< 0.001	-2.17
**GM (R) Elast.**	1.05 ± 0.12	1.10 ± 0.16	0.308	-0.35
**GM (L) Elast.**	1.12 ± 0.13	1.06 ± 0.12	0.185	0.46
**GL (R) Tone**	14.53 ± 1.03	18.57 ± 1.63	< 0.001	-2.95
**GL (L) Tone**	14.47 ± 1.17	18.95 ± 2.17	< 0.001	-2.55
**GL (R) Stiff.**	260.18 ± 40.24	357.06 ± 50.60	< 0.001	-2.11
**GL (L) Stiff.**	258.47 ± 40.94	364.17 ± 72.99	< 0.001	-1.77
**GL (R) Elast.**	1.00 ± 0.11	1.02 ± 0.12	0.683	-0.14
**GL (L) Elast.**	0.99 ± 0.12	0.97 ± 0.14	0.713	0.12
**TA (R) Tone**	17.40 ± 1.74	22.43 ± 2.75	< 0.001	-2.17
**TA (L) Tone**	17.74 ± 1.37	22.51 ± 2.05	< 0.001	-2.73
**TA (R) Stiff.**	395.41 ± 88.93	495.22 ± 110.87	0.006	-0.99
**TA (L) Stiff.**	401.06 ± 80.34	508.28 ± 97.47	0.001	-1.20
**TA (R) Elast.**	0.89 ± 0.12	0.78 ± 0.22	0.088	0.59
**TA (L) Elast.**	0.88 ± 0.15	0.77 ± 0.12	0.022	0.82
**Q (R) Tone**	13.50 ± 0.78	15.79 ± 1.26	< 0.001	-2.17
**Q (L) Tone**	13.71 ± 0.70	15.96 ± 1.53	< 0.001	-1.87
**Q (R) Stiff.**	230.71 ± 29.98	282.94 ± 29.26	< 0.001	-1.76
**Q (L) Stiff.**	226.65 ± 24.89	290.44 ± 39.08	< 0.001	-1.93
**Q (R) Elast.**	1.20 ± 0.22	1.33 ± 0.27	0.123	-0.53
**Q (L) Elast.**	1.17 ± 0.18	1.30 ± 0.27	0.094	-0.58
**H (R) Tone**	13.29 ± 1.27	16.20 ± 1.71	< 0.001	-1.92
**H (L) Tone**	13.37 ± 1.16	16.32 ± 1.22	< 0.001	-2.47
**H (R) Stiff.**	204.29 ± 40.40	298.89 ± 53.94	< 0.001	-1.98
**H (L) Stiff.**	204.35 ± 34.77	305.89 ± 30.84	< 0.001	-3.10
**H (R) Elast.**	1.02 ± 0.15	1.16 ± 0.17	0.021	-0.82
**H (L) Elast.**	1.10 ± 0.18	1.23 ± 0.18	0.033	-0.75
**Strength Outcomes**				
**Grip_Dom**	27.67 ± 3.48	42.63 ± 6.08	< 0.001	-3.00
**Grip_NonDom**	26.33 ± 4.03	39.28 ± 6.02	< 0.001	-2.51
**Leg_Dom**	234.06 ± 14.98	277.82 ± 16.53	< 0.001	-2.77
**Leg_NonDom**	237.26 ± 16.05	281.14 ± 16.46	< 0.001	-2.70
**Leg_Double**	252.36 ± 13.32	298.14 ± 5.40	< 0.001	-4.55
**Back_Str**	243.18 ± 16.67	294.81 ± 9.83	< 0.001	-3.80
**Shoulder_IR_Max**	7.99 ± 2.48	16.16 ± 5.44	< 0.001	-1.91
**Shoulder_IR_Avg**	3.55 ± 2.59	5.91 ± 2.32	0.008	-0.96
**Balance Outcomes**				
**EC_Static_Tot**	768.41 ± 785.61	1578.61 ± 642.80	0.002	-1.13
**EC_Dynamic_Tot**	3549.41 ± 1234.93	3491.44 ± 636.58	0.864	0.06
**EO_Static_Tot**	240.88 ± 250.01	287.00 ± 120.61	0.498	-0.24
**EO_Dynamic_Tot**	983.29 ± 366.29	1039.72 ± 267.14	0.608	-0.18

Table S1. Descriptive statistics, between-sex comparisons, and effect sizes (Cohen's d) for myotonic, strength, and balance variables.
